# Supplementary material for: Viscoelastic characteristics of the canine cranial cruciate ligament complex at slow strain rates
Source: PeerJ. 2020 Dec 22;8:e10635. doi: 10.7717/peerj.10635 (PMC7761198; doi:10.7717/peerj.10635)
Supplement: Supplemental Information 1 [file peerj-08-10635-s001.docx]

**Supplementary Materials**

Tables

**Table S1**: P-values obtained from Bonferroni post-hoc statistical analysis on ligament lengths at different planes.

| **Cranial and Caudal Planes** | **Cranial and Medial Planes** | **Cranial and Lateral Planes** | **Caudal and Medial Planes** | **Caudal and Lateral Planes** | **Medial and Lateral Planes** |
| --- | --- | --- | --- | --- | --- |
| $6.74\times{10}^{-7}$ | $9.96\times{10}^{-3}$ | $2.25\times{10}^{-2}$ | $1.49\times{10}^{-5}$ | $1.41\times{10}^{-4}$ | $9.07\times{10}^{-1}$ |

**Table S2**: The table below shows the stored energy under loading and unloading stress-strain curves and hysteresis for the cranial cruciate ligaments (CCLs) at three strain rates. Stored energy in the CCLs determined by summing the area under stress-strain curves during loading and unloading tests. Subsequently, energy dissipated (hysteresis) values were determined by finding the difference between stored energy during loading and unloading tests.

| **Ascending Tests** | | | | | | | | | **Descending Tests** | | | | | | | |
| --- | --- | --- | --- | --- | --- | --- | --- | --- | --- | --- | --- | --- | --- | --- | --- | --- |
| **Strain rates (%/min)** | **Stored Energy (MPa) - Loading** | | | | | | | | **Stored Energy (MPa) - Loading** | | | | | | | |
|  | **CCL 1** | **CCL 2** | **CCL 3** | **CCL 4** | **CCL 5** | **CCL 6** | **Average** | **SD** | **CCL 1** | **CCL 2** | **CCL 3** | **CCL 4** | **CCL 5** | **CCL 6** | **Average** | **SD** |
| **0.1** | 0.015 | 0.0116 | 0.0056 | 0.0156 | 0.014 | 0.0085 | 0.0117 | 0.004 | 0.0298 | 0.0066 | 0.0176 | 0.0108 | 0.0193 | 0.0136 | 0.0163 | 0.0081 |
| **1** | 0.0134 | 0.0096 | 0.0046 | 0.0133 | 0.0119 | 0.0072 | 0.01 | 0.0035 | 0.0295 | 0.0065 | 0.0167 | 0.0115 | 0.0168 | 0.0128 | 0.0156 | 0.0078 |
| **10** | 0.0127 | 0.0091 | 0.0044 | 0.0128 | 0.0114 | 0.007 | 0.0096 | 0.0034 | 0.0288 | 0.0064 | 0.0164 | 0.0113 | 0.0164 | 0.0125 | 0.0153 | 0.0076 |
|  | | | | | | | | |  |  |  |  |  |  |  |  |
| **Strain rates (%/min)** | **Stored Energy (MPa) - Unloading** | | | | | | | | **Stored Energy (MPa) - Unloading** | | | | | | | |
|  | **CCL 1** | **CCL 2** | **CCL 3** | **CCL 4** | **CCL 5** | **CCL 6** | **Average** | **SD** | **CCL 1** | **CCL 2** | **CCL 3** | **CCL 4** | **CCL 5** | **CCL 6** | **Average** | **SD** |
| **0.1** | 0.0126 | 0.0079 | 0.0037 | 0.0108 | 0.0103 | 0.0059 | 0.0085 | 0.0033 | 0.0233 | 0.0053 | 0.0138 | 0.008 | 0.0131 | 0.0098 | 0.0122 | 0.0063 |
| **1** | 0.0114 | 0.0075 | 0.0035 | 0.0105 | 0.0096 | 0.0057 | 0.008 | 0.003 | 0.0214 | 0.0054 | 0.0127 | 0.0079 | 0.0116 | 0.0096 | 0.0114 | 0.0056 |
| **10** | 0.0112 | 0.0075 | 0.0036 | 0.0104 | 0.0096 | 0.0057 | 0.008 | 0.0029 | 0.0213 | 0.0055 | 0.0125 | 0.0083 | 0.0121 | 0.01 | 0.0116 | 0.0054 |
|  | | | | | | | | |  |  |  |  |  |  |  |  |
| **Strain rates (%/min)** | **Dissipated Energy (MPa)** | | | | | | | | **Dissipated Energy (MPa)** | | | | | | | |
|  | **CCL 1** | **CCL 2** | **CCL 3** | **CCL 4** | **CCL 5** | **CCL 6** | **Average** | **SD** | **CCL 1** | **CCL 2** | **CCL 3** | **CCL 4** | **CCL 5** | **CCL 6** | **Average** | **SD** |
| **0.1** | 0.0025 | 0.0037 | 0.0019 | 0.0047 | 0.0038 | 0.0026 | 0.0032 | 0.0011 | 0.0065 | 0.0012 | 0.0037 | 0.0028 | 0.0062 | 0.0038 | 0.004 | 0.002 |
| **1** | 0.0019 | 0.0021 | 0.0011 | 0.0028 | 0.0023 | 0.0015 | 0.002 | 0.0006 | 0.0081 | 0.0011 | 0.004 | 0.0036 | 0.0052 | 0.0031 | 0.0042 | 0.0023 |
| **10** | 0.0016 | 0.0016 | 0.0009 | 0.0024 | 0.0018 | 0.0012 | 0.0016 | 0.0005 | 0.0074 | 0.0009 | 0.0038 | 0.0029 | 0.0043 | 0.0026 | 0.0037 | 0.0022 |

Figures

| 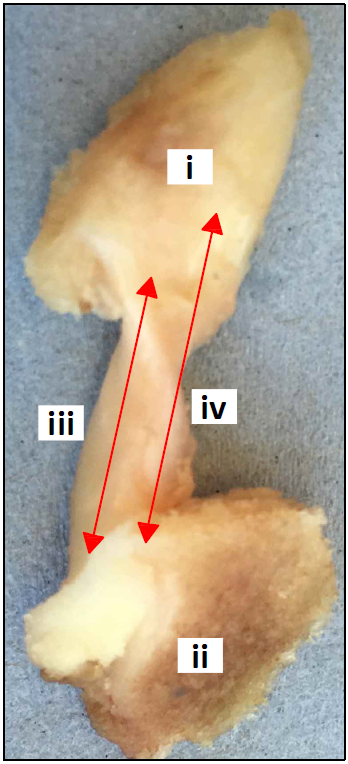  **(a)** | 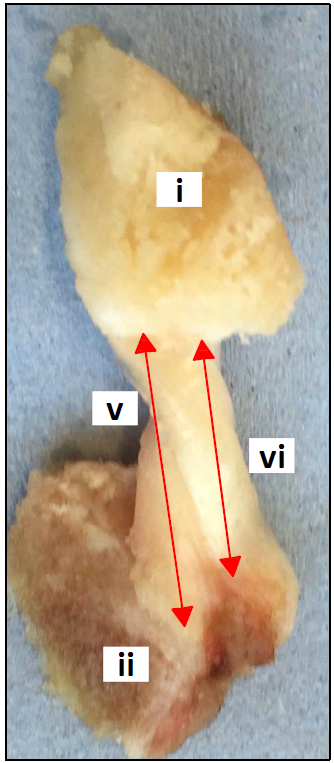  **(b)** |
| --- | --- |

Fig. S1. The cranial cruciate ligament (CCL) from a right canine stifle joint from the (a) cranial, and (b) caudal views of the ligament. The length of the CCL, attached to the (i) femur and (ii) tibia was measured at four different planes: (iii) lateral, (iv) cranial, (v) medial and (vi) caudal.

| **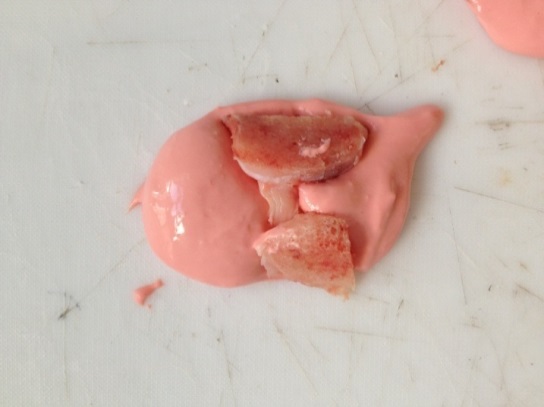**  **(a)**  **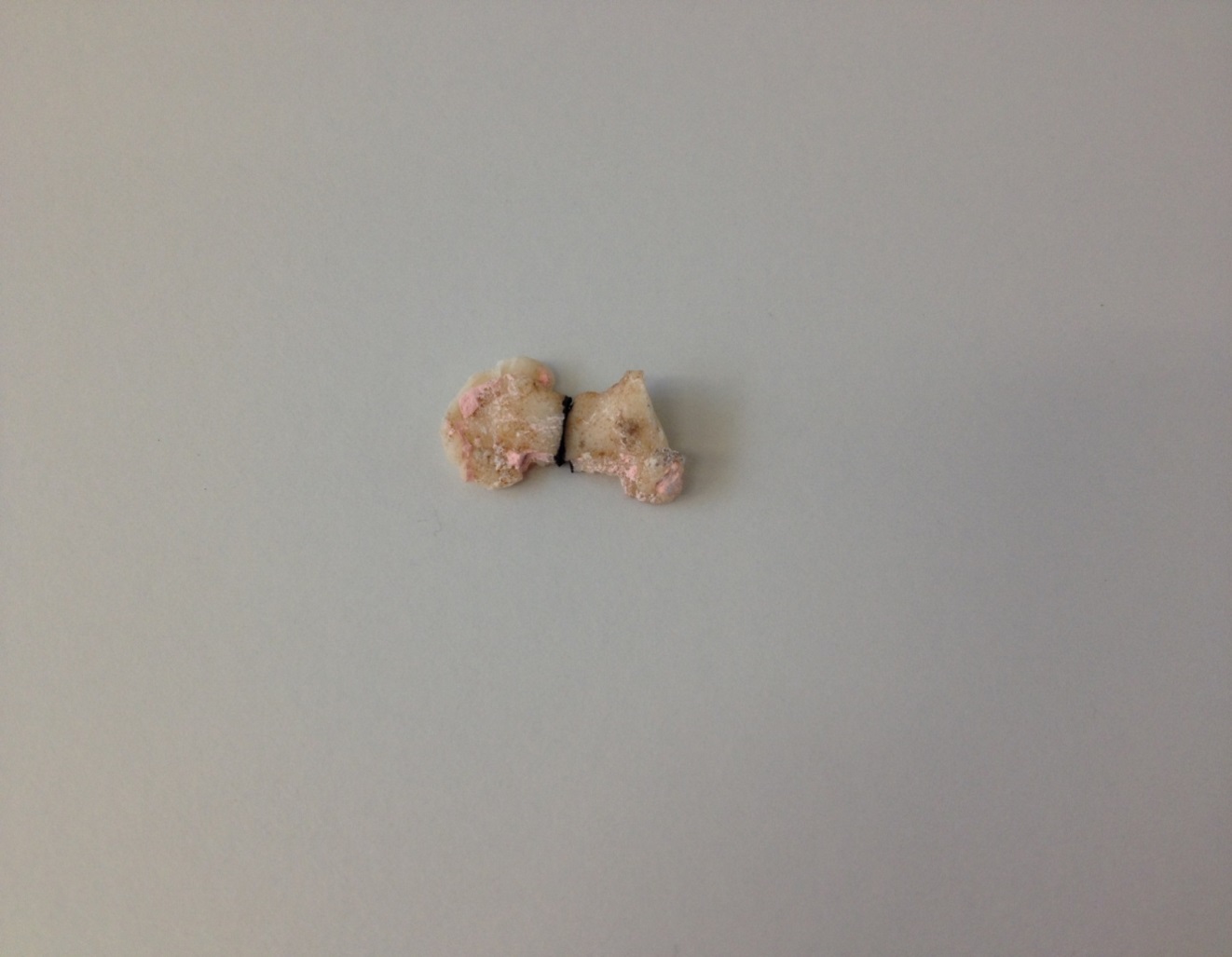**  **(c)** | **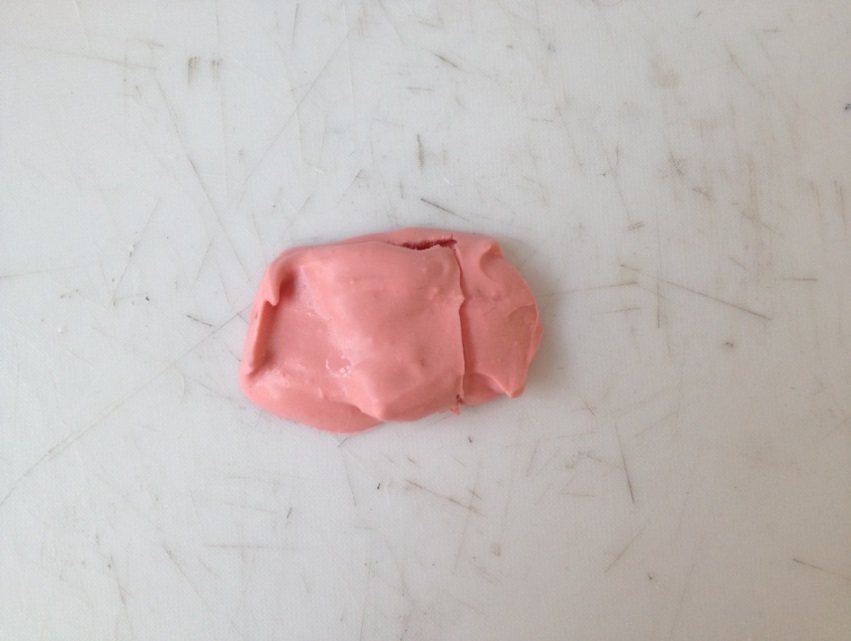**  **(b)**  **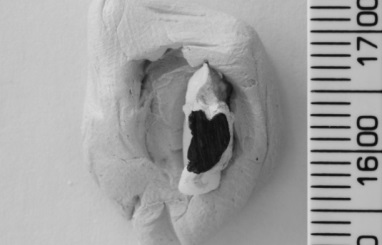**  **(d)** |
| --- | --- |

Fig. S2. The middle cross-sectional area (CSA) of the cranial cruciate ligament (CCL) was estimated by firstly creating an alginate paste and (a) place the paste around the CCL until it was set. (b) Subsequently, the CCL was removed from the set paste leaving behind an alginate mould for the ligament. (c) Poly methyl methacrylate (PMMA) was injected into the mould to create replica model of the CCL. The replica was cut into two in the middle and (d) the surface of the replica showing middle CSA was coloured in black paint. The black surface area of the replica was then measured using ImageJ (a public domain Java image processing program).

| **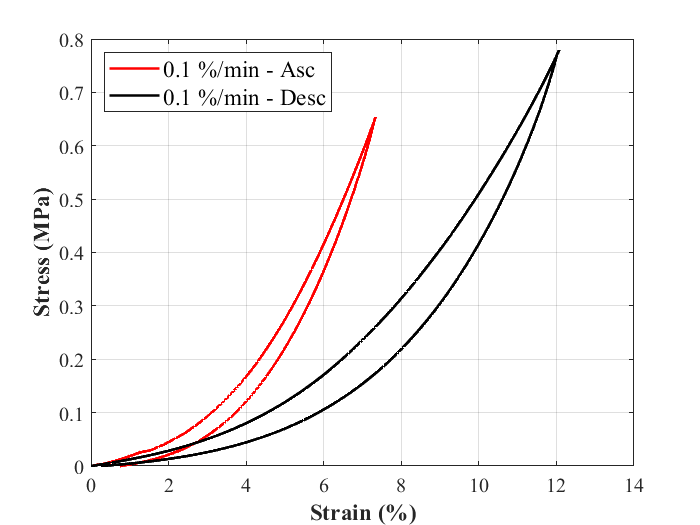**  **CCL 1** | **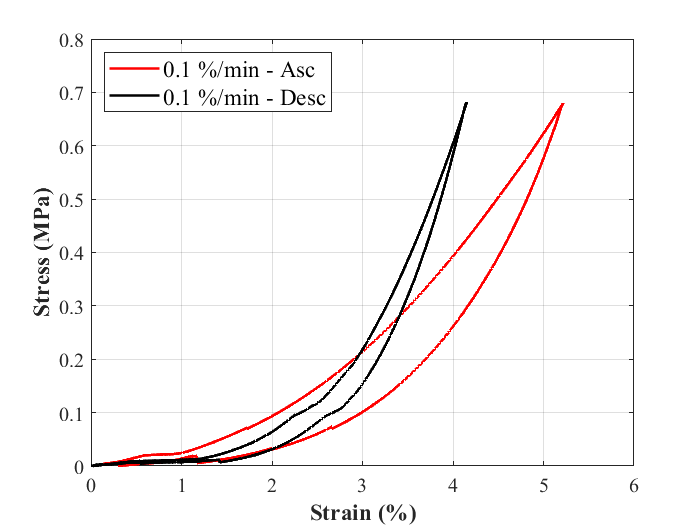**  **CCL 2** | **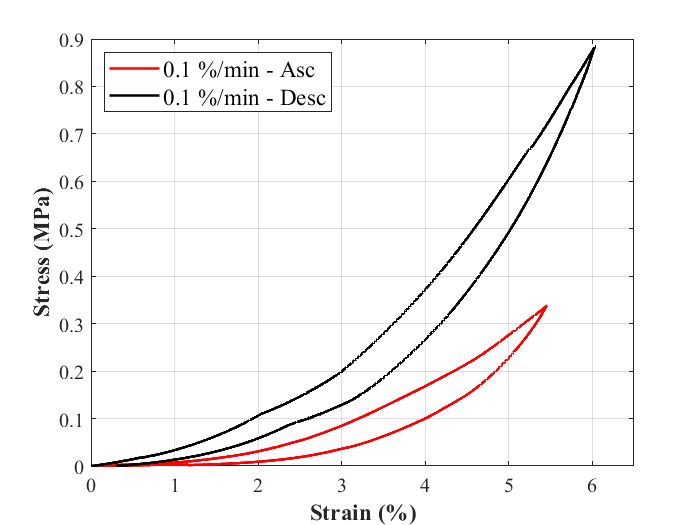**  **CCL 3** |
| --- | --- | --- |
| **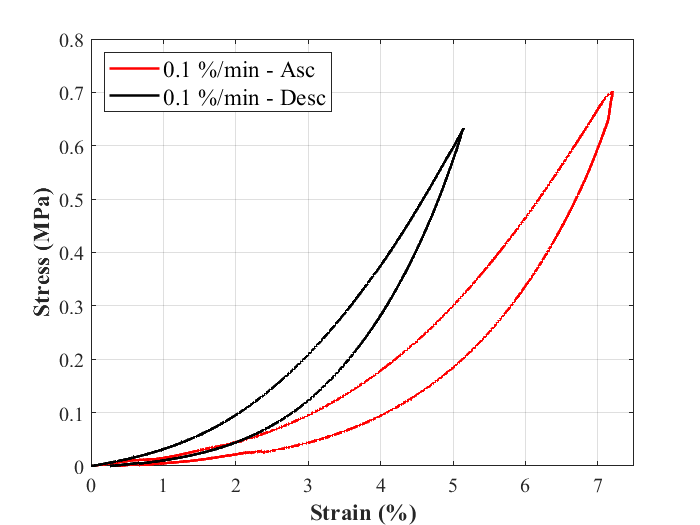**  **CCL 4** | **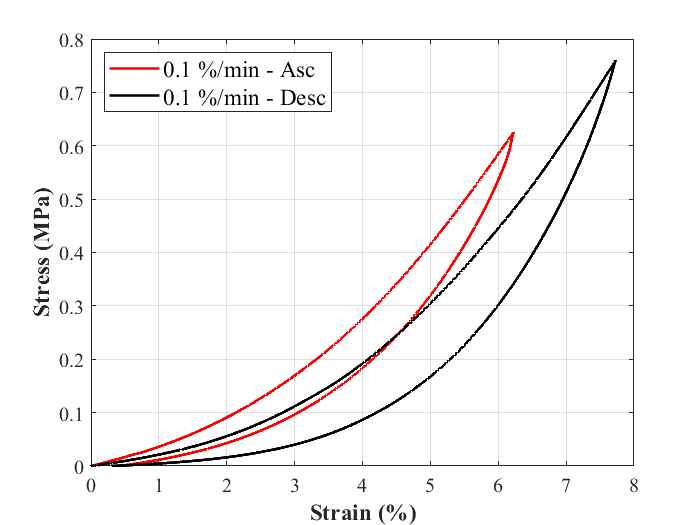**  **CCL 5** | **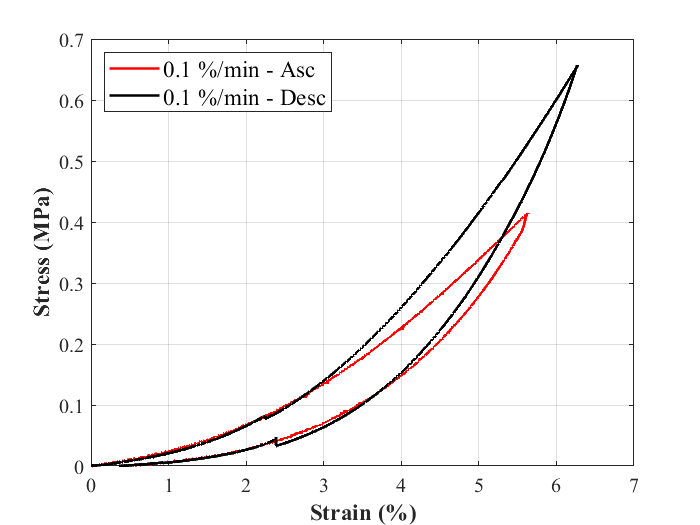**  **CCL 6** |

**Fig. S3.** The loading and unloading stress-strain characteristics of individual canine cranial cruciate ligaments (CCL) during the ascending (Asc) and descending (Desc) tests at 0.1%/min strain rate.

| 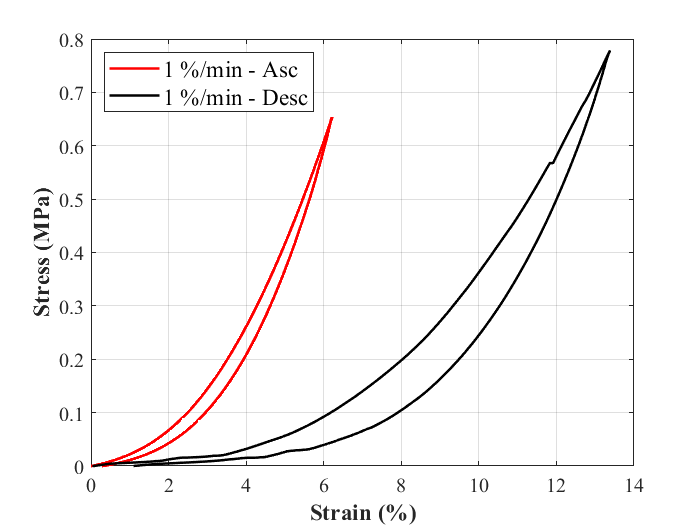  **CCL 1** | 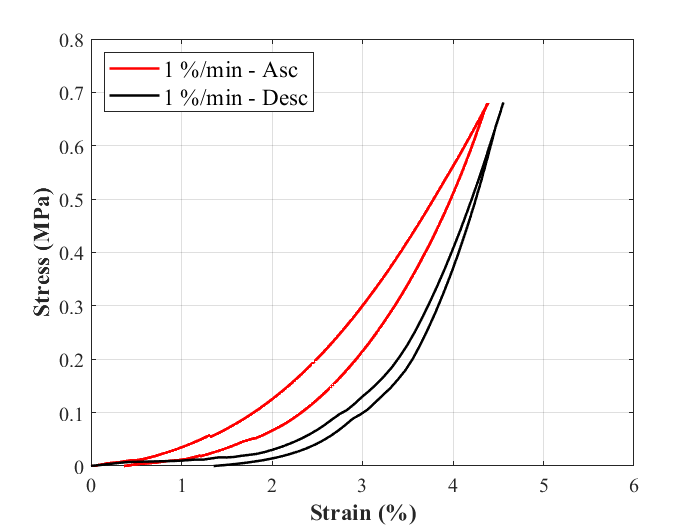  **CCL 2** | 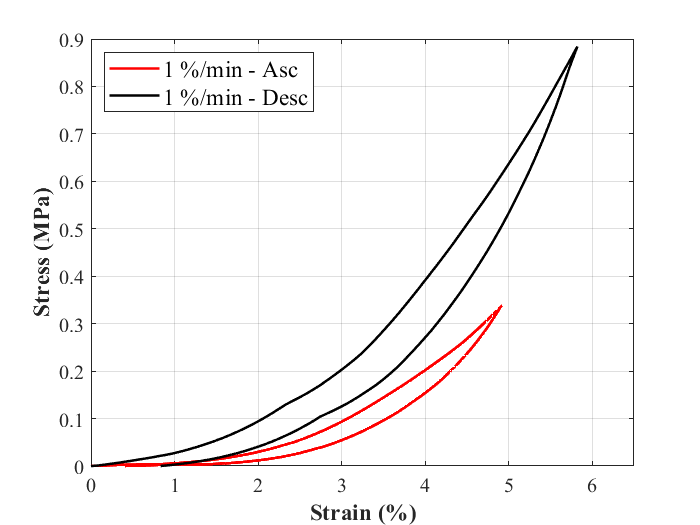  **CCL 3** |
| --- | --- | --- |
| 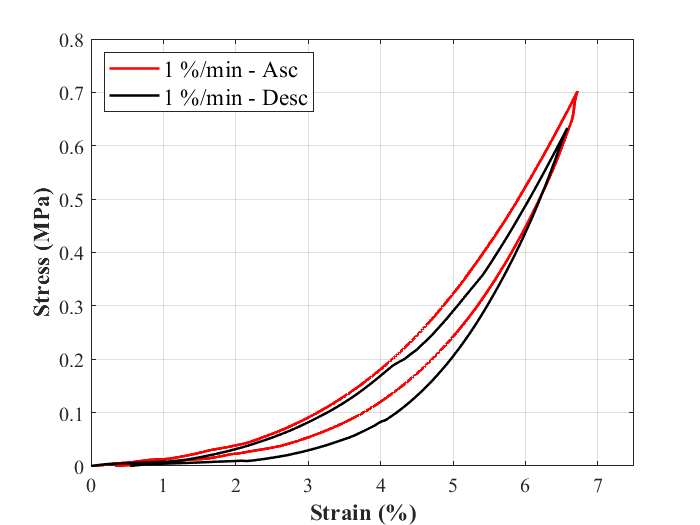  **CCL 4** | 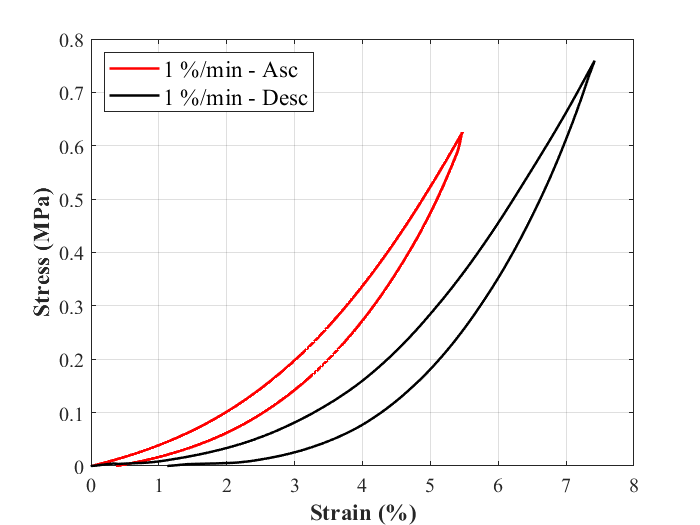  **CCL 5** | 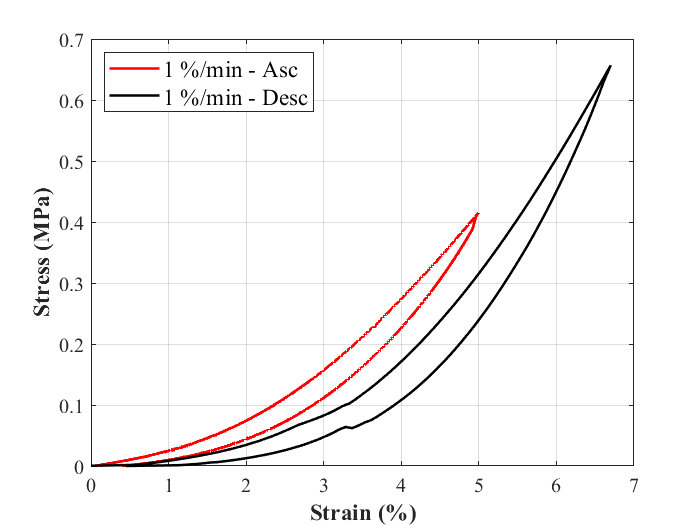  **CCL 6** |

**Fig. S4.** The loading and unloading stress-strain characteristics of individual canine cranial cruciate ligaments (CCL) during the ascending (Asc) and descending (Desc) tests at 1%/min strain rate.

| 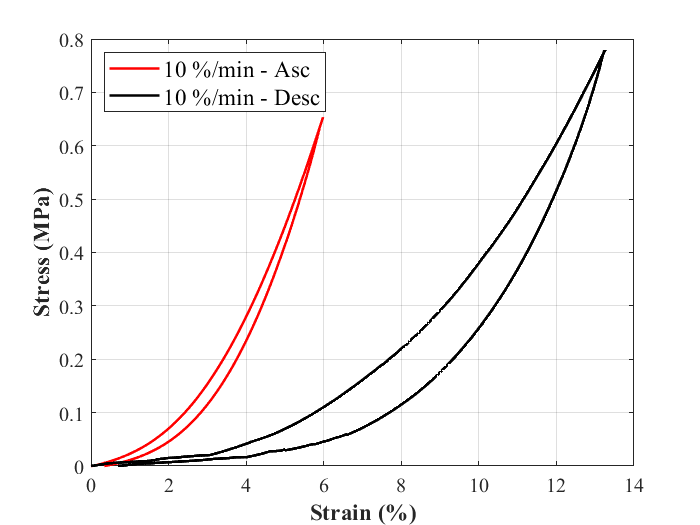  **CCL 1** | 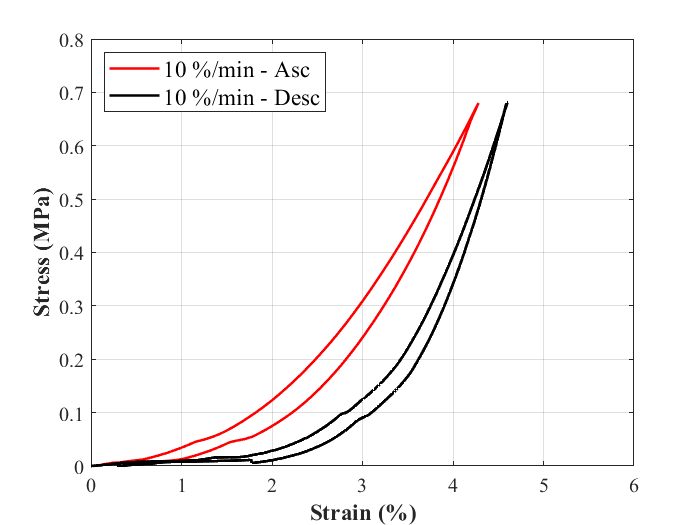  **CCL 2** | 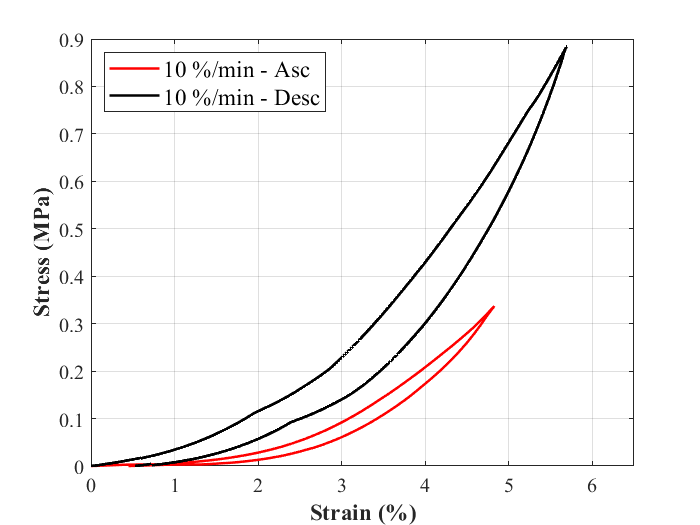  **CCL 3** |
| --- | --- | --- |
| 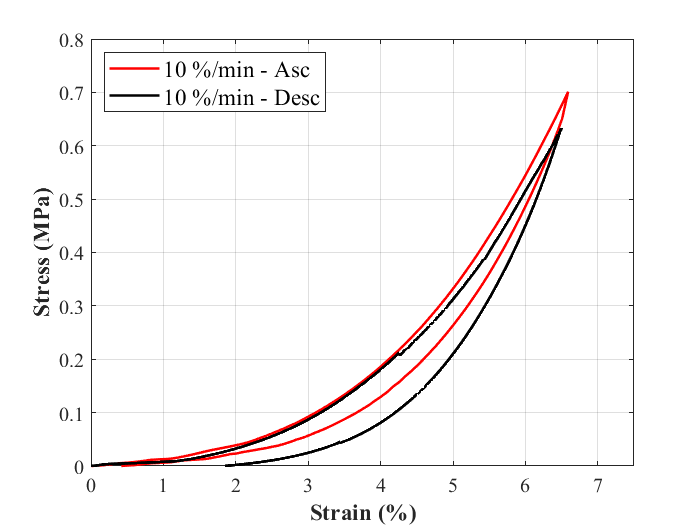  **CCL 4** | 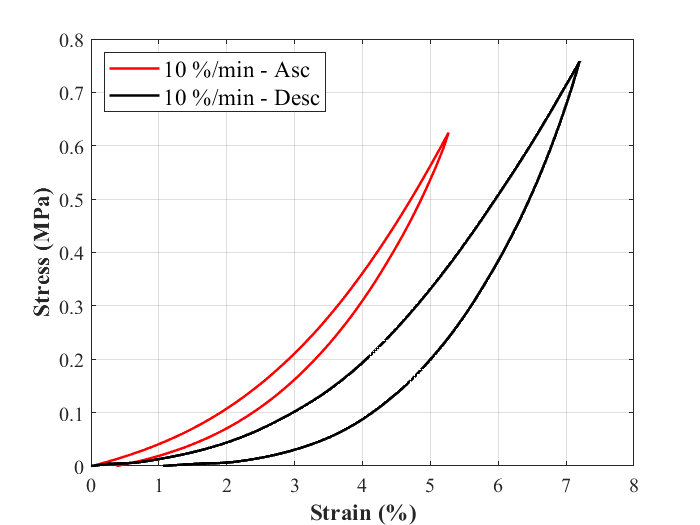  **CCL 5** | 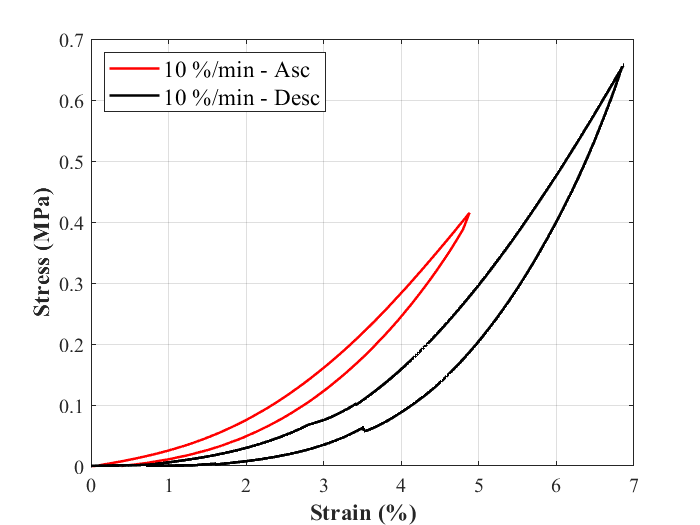  **CCL 6** |

**Fig. S5.** The loading and unloading stress-strain characteristics of individual canine cranial cruciate ligaments (CCL) during the ascending (Asc) and descending (Desc) tests at 10%/min strain rate.


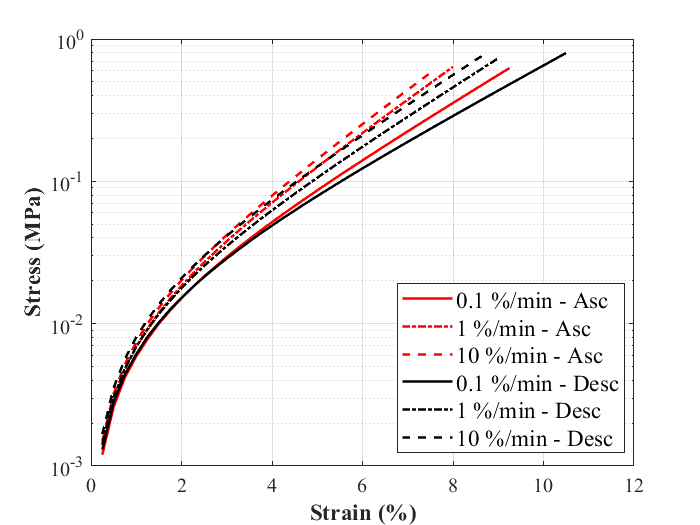


**Fig. S6.** An example of the loading part of stress-strain curve of a canine cranial cruciate ligament (CCL) at varying strain rates on a semi-log scale graph. The tensile characteristics of the CCLs was investigated following ascending (Asc) and descending (Desc) protocols at 0.1, 1 and 10 %/min strain rates.
